# Selection and evaluation of reference genes for quantitative real-time polymerase chain reaction normalization in *Pieris melete* (Lepidoptera, Pieridae)

**DOI:** 10.1093/jisesa/ieaf108

**Published:** 2025-12-22

**Authors:** Ting Jiang, Xiangya Liu, Ning Zou, Wanna Zhang, Yingchuan Peng, Haijun Xiao

**Affiliations:** Jiangxi Cash Crops Research Institute/Jiangxi Provincial Key Laboratory of Plantation and High Valued Utilization of Specialty Fruit Tree and Tea, Nanchang, China; Institute of Entomology, Jiangxi Agricultural University, Nanchang, China; Institute of Entomology, Jiangxi Agricultural University, Nanchang, China; Jiangxi Grain and Oil Science and Technology Innovation and Material Reserve Center, Nangchang, China; Institute of Entomology, Jiangxi Agricultural University, Nanchang, China; Institute of Entomology, Jiangxi Agricultural University, Nanchang, China; Institute of Entomology, Jiangxi Agricultural University, Nanchang, China; Institute of Entomology, Jiangxi Agricultural University, Nanchang, China; School of Grassland Science, Beijing Forestry University, Beijing, China

**Keywords:** housekeeping genes, expression stability evaluation, algorithmic concordance, normalization strategy, cabbage butterfly

## Abstract

Accurate normalization of gene expression data in real-time quantitative polymerase chain reaction (RT-qPCR) relies on the identification of stably expressed reference genes, which remains uncharacterized in *Pieris melete* Ménétriés, a cruciferous crop pest with agricultural significance. This study systematically evaluated eight candidate reference genes (*GAPDH*, *α-tub*, *β-actin*, *18S*, *β-tub*, *EF1α*, *RPL27*, *RPS15*) across four experimental conditions: developmental stages, tissues, temperature stresses, and diapause stages. Stability rankings were determined using four algorithms (geNorm, NormFinder, BestKeeper, Δ*Ct* method) integrated via RefFinder. Results revealed that at different condition-specific stability patterns, *RPL27* and *EF1α* were optimal for developmental stages, *RPL27* paired with *18S* were suitable for tissue analyses; *EF1α* and *α-tub* were stable at different temperature stresses, and *RPL27* combined with *RPS15* were stably expressed during diapause. Pairwise variation analysis confirmed that dual reference genes sufficiently enhanced normalization accuracy. This work provides the validated reference genes panel for *P. melete*, addressing a critical gap in molecular studies of this pest and ensuring robust gene expression analyses for future research on diapause regulation and pest control strategies.

## Introduction

Real-time quantitative reverse transcription–polymerase chain reaction (RT-qPCR/qPCR) stands as a widely adopted tool in molecular biological research, enabling the quantification of target gene expression and the quantitative analysis of functional gene expression patterns (Bustin et al. 2009). It boasts multiple strengths compared with conventional methods for measuring gene expression: simplicity, reproducibility, high efficiency, and strong sensitivity and specificity even for target genes at low abundance (Shakeel et al. 2018, Shen et al. 2024). Nonetheless, its precision is impacted by numerous unavoidable factors, including RNA integrity, cDNA synthesis efficiency, amplification performance, and pipetting inconsistencies (Valasek and Repa 2005, Udvardi et al. 2008, Lü et al. 2018). To ensure accurate gene quantification, normalization thus becomes a vital step in qPCR experiments. This process entails evaluating and selecting one or more reference genes with stable expression to offset errors arising from experimental variability (Bustin et al. 2009, Ruijter et al. 2021).

Reference genes are typically housekeeping genes (HKGs) like *GAPDH*, *18S*, *α-tub*, *β-actin*, whose expression is conventionally assumed to remain constant under diverse treatment conditions (Thellin et al. 1999, Butte et al. 2001). Yet this assumption does not hold universally. Instead, numerous studies have indicated that the expression of commonly used reference genes can fluctuate significantly across different treatments in a specific organism (Sagri, et al. 2017, Lü et al. 2018, Bansal et al. 2023, Wang et al. 2023). Consequently, using reference genes for normalization without first verifying their expression stability under the specific experimental conditions may lead to inaccurate results and misleading conclusions (Majerowicz et al. 2011, Zhu et al. 2014, Luo et al. 2018). Additionally, the number of reference genes utilized for normalization can also influence the accuracy of gene expression quantification (Derveaux et al. 2010). Hence, a systematic and context-specific investigation into valid reference genes—tailored to each organism and its respective experimental conditions—is strongly recommended.

Prior studies on insects have identified optimal reference genes based on their expression stability under specific experimental conditions, for example, the cotton bollworm *Helicoverpa armigera* (Zhang et al. 2015), the rice stem borer *Chilo suppressalis* (Xu et al. 2017), the Asian ladybird *Harmonia axyridis* (Yang et al. 2018), the fall armyworm *Spodoptera frugiperda* (Shu et al. 2021), the tobacco cutworm *Spodoptera litura* (Wu et al. 2024), the ‌lorey armyworm *Mythimna loreyi* (Wang et al. 2024), etc. These studies have indicated that the expression level of several reference genes is condition-specific and accordingly, strongly emphasize the necessity of evaluating appropriate reference genes for accurate and reliable normalization on a case-by-case basis, even for the same species.

The cabbage butterfly *Pieris melete* Ménétriés (Lepidoptera, Pieridae) is a major pest for the cruciferous in Jiangxi Province in China, and has the biological characteristic of summer and winter diapause as a pupa (Xue et al. 1997). *P. melete* diapause has been systematically studied over the past decades from biological physiological, and ecological perspectives, under laboratory and natural field conditions, and showing high adaptability to local seasonal environmental challenges (Xiao et al. 2012) In recent years, a significant number of genes essential to insect diapause processes, including heat shock proteins, photoperiodism, hormone metabolism, defense response, and circadian clock genes, were screened from the transcriptome data of *P. melete* (Jiang et al. 2021). Future investigations will focus on targeted expression analysis of these genes using RT-qPCR, a highly appropriate method for this purpose. However, to date, the stability of reference genes under abiotic or biotic conditions has yet to be systematically evaluated and validated. Currently, the assembly and analysis of the transcriptome data for *P. melete* have been completed, providing comprehensive genetic information, which enhances the accuracy and reliability of the screening for reference genes.

Therefore, in this study, we assessed the stability of eight candidate HKGs in insects for use as internal references in RT-qPCR for normalization in *P. melete: GAPDH*, *α-tub*, *β-actin*, *18S*, *β-tub*, *EF1a*, *RPL27*, *RPS15*. The most appropriate reference genes were tested with respect to different developmental stages, tissues, temperatures, and especially under different diapause stages using RefFinder (Xie et al. 2012, Xie et al. 2023), which integrates four different statistical algorithms (geNorm) (Vandesompele et al. 2002), NormFinder (Andersen et al. 2004), BestKeeper (Pfaffl et al. 2004), and the comparative Δ*Ct* method (Silver et al. 2006). This is the first study to investigate appropriate reference genes in this pest insect. Our results will serve as a guide for accurate relative quantification of candidate gene expression in *P. melete* across a range of experimental conditions, and are important for application of RT-qPCR in future experiments of novel tactics to control this species.

## Materials and Methods

### Insects

The insects were reared according to the method of Xiao et al. (2008). Eggs of *P. melete* were obtained from the Experimental Station of Entomology department in Jiangxi Agricultural University, Nanchang, China. The insects were maintained in the laboratory at 20 ± 1 °C under a photoperiod of 14:10 h light (L): dark (D) and 75% relative humidity, and were fed with fresh *Raphanus* leaves.

### Experimental Treatments

The effect of developmental stages, tissues, temperatures and diapause stages on reference gene expression were evaluated in the *P. melete*. Samples were collected under various conditions and stored in 1.5 mL centrifuge tubes. The samples comprised the following: (i) Different developmental stages: Approximately 50 eggs; 30, 15, 5, 1, and 1 of the first to fifth-instar larvae, respectively; and one individual each of the prepupal, one-day-old pupal, and adult (collected within two days after eclosion) stages were collected. (ii) Larval tissues: The head, epidermis, fat body, midgut, and Malpighian tubules were dissected from nine fifth-instar larvae. Prior to dissection, the larvae were rinsed with ice-cold phosphate-buffered saline to remove surface contaminants. All dissections were performed under a binocular microscope using sterilized scalpels and tweezers. (iii) Temperature treatments: Groups of 10 fourth-instar larvae of similar size were exposed to 0 °C (cold shock), 20 °C (control), 40 °C (heat shock) for 2 h. (iv) Diapause stages: In particular, we collected pupae undergoing summer diapause, which was induced under a long-day photoperiod (14:10 h L:D) at 20 ± 1 °C and 75% humidity. Samples of 10 individuals each were taken at the early, middle, and late stages of diapause. All the samples were immediately frozen in liquid nitrogen before storage at −80 °C before RNA extraction. Each experiment was replicated three times.

### RNA Extraction and cDNA Preparation

According to the manufacturer’s instructions, total RNA from each sample was isolated using Trizol reagent (Invitrogen, United States). The RNA concentrations and purity were determined by Nano Photometer micro-volume spectrophotometer and 1% agarose gel. Finally, first-strand cDNA was synthesized from 1,000 ng of total RNA in accordance with the manual FastKing RT Kit (With gDNase) (Tiangen, Beijing, China) and stored at −20 °C.

### Identification of Candidate Reference Genes

Based on previous studies of reference genes from other lepidoptera species, eight candidate reference genes, namely *EF1α*, *GAPDH*, *α-tub*, *β-tub*, *β-actin*, *RPL27*, *RPS15*, *and 18S*, were selected from transcriptome database of *P. melete* (GenBank accession number: PRJNA625900). A local BLAST search against the transcriptome of *P. melete* was performed to find homologs of these candidate genes. Then, homology of the sequences was assessed by performing a BLAST analysis on the NCBI platform. Details of the alignment results are shown in Supplementary Table S1 (Supplementary Material S1). Furthermore, the open reading frames of these genes were verified by PCR amplification using specific primers. The transcript sequences of these genes are provided in Supplementary Material S2. Pairs of gene-specific primers based on recently sequenced transcriptomes used for RT-qPCR were designed with Beacon Designer 8.0 (Bio-Rad, United States). The specification information of primers included in the study is given in Table 1.

**Table 1.** RT-qPCR primer information of candidate reference genes in *Pieris melete*

Genes	Full name	Primer sequences (5′ to 3′)	Product length (bp)	Amplification efficiency (*E*) (%)	Regression coefficient (*R* ^2^)
*α-tub*	Alpha tubulin	F: GTTGGTCTGGAACTCGGTAAGAT R: GAACAGGCTTATCGGTCAAATCG	94	99.68	0.9998
*β-actin*	Beta actin	F: GGCGTCTATGAAATGTGATTCGG R: AGACCTGGAATCATCGCTAATCC	87	104.61	0.9992
*EF1a*	Elongation factor 1 alpha	F: GGATGGTTCAACACAATGACCTG R: GAACGTCTCTGTCAAGGAATTGC	111	106.09	0.9929
*RPL27*	Ribosomal protein L27	F: CTCTTGTGAACTTTCCTGGGGTA R: GTCGTTAAGACCTATGACGAGGG	95	103.90	0.9991
*RPS15*	Ribosomal protein S15	F: CGCCATTGGTTTCCTCTTAAGAC R: CTTCCGAGGGGTTGACTTAGATC	118	98.73	0.9987
*18S rRNA*	18S ribosomal RNA	F: AATGCCGCTTGAATATTTCG R: TTTCGCTGATGTTCGTCTTG	167	96.42	0.9975
*β-tub*	Beta tubulin	F: CAGCTGTCCGGGAAATCTTAAAC R: CTTCGGTTACAGACCCCAACTTA	96	98.52	0.9958
*GAPDH*	Glyceraldehyde-3-phosphate dehydrogenase	F: TGGATGCCTAGTAGTCAAC R: TGCCTTATCAATGGTGGTA	133	93.47	0.9986

### Quantitative Real-Time PCR

RT-qPCR analyses were carried out using CFX96 Real-Time PCR Detection System (Bio-Rad, United States) in which amplification, detection, and analysis steps were combined. A total of 20 μL reaction mixture volume for each sample was configured, which contained 10 μL TB Green Premix Ex Taq II (Takara, Japan), 1 μL (10 μM) each of the forward and reverse primer, 2 μL cDNA template, and 6 μL RNase-free water. Briefly, the amplification was conducted according to the following cycling parameters: 95 °C for 30 s, followed by 40 cycles of 95 °C for 5 s and 60 °C for 30 s and a final melting curve analysis to further confirm the specificity of amplified product for each sample from 65 °C to 95 °C. To select the optimal gene-specific primer of each candidate reference gene, a 5-point standard curve for each primer pair was constructed with a 5-fold dilution series of cDNA as a template by using the linear regression model. The amplification efficiency (*E*) and correlation coefficient values (*R*^2^) were calculated from standard curves according to the formula: *E* (%) = (10 ^[−1/slope]^ − 1) ×100 (Pfaffl 2001). All RT-qPCR assays were completed with three biological replicates and three technical replicates.

### Stability Analysis of Reference Genes Expression

The expression stability of candidate HKGs were evaluated with an online analysis software RefFinder (http://www.heartcure.com.au/reffinder/) which included Genorm, NormFinder, BestKeeper, and the comparative Δ*Ct* method. The four different methods focus on different factors. Genorm evaluates the most stable reference genes by calculating the gene expression stability value “*M*”, with a lower value indicating a more stable expression. Furthermore, Genorm also determines the optimal number of reference genes for normalization. The pairwise variations *Vn/n + *1 (*n* is the number of reference genes) between two sequential normalization factors, <0.15 means that no additional genes are required for normalization. NormFinder uses a model-based approach to rank the candidate reference genes by their stability value. Candidate gene with the lowest value should be the most stably expressed reference gene. In BestKeeper, *P* values, the standard deviation (SD) and coefficient of variation values based on *Ct* values were the decisive factors that determined the most appropriate criteria. The comparative Δ*Ct* method relies on the relative expression of pairwise genes within each sample. Candidate reference genes with smaller Δ*Ct* variability were considered as the most stable. Finally, we comprehensively compared and ranked the tested candidate genes based on the geomean values of the above results from the four different algorithms by using the web-based tool RefFinder.

## Results

### Primer Specificity and Efficiency

Before evaluating the suitability of the reference genes, the specificity and efficiency of PCR amplification were first validated. The PCR amplification for each designed primer pair showed a single band of the anticipated size, as confirmed by a 1.0% agarose gel electrophoresis (Supplementary Fig. S1), and a solitary peak in the melting curve analysis (Supplementary Fig. S2). Additionally, the RT-qPCR amplification of eight candidate gene transcripts from a serial diluted cDNA template of dilution range (1, 1:5, 1:25, 1:125, 1:625) resulted in amplification efficiency (*E*) ranging from 93.47% (*GAPDH*) to 106.09% (*EF1α*), with high linear regression coefficients *R^2^* ≥ 0.990 (Table 1). Collectively, these results demonstrate that the designed primers met the requirements of fluorescence quantitative analysis and were suitable for subsequent quantitative assays.

### Expression Profiles of Candidate Reference Genes

**Fig. 1.**
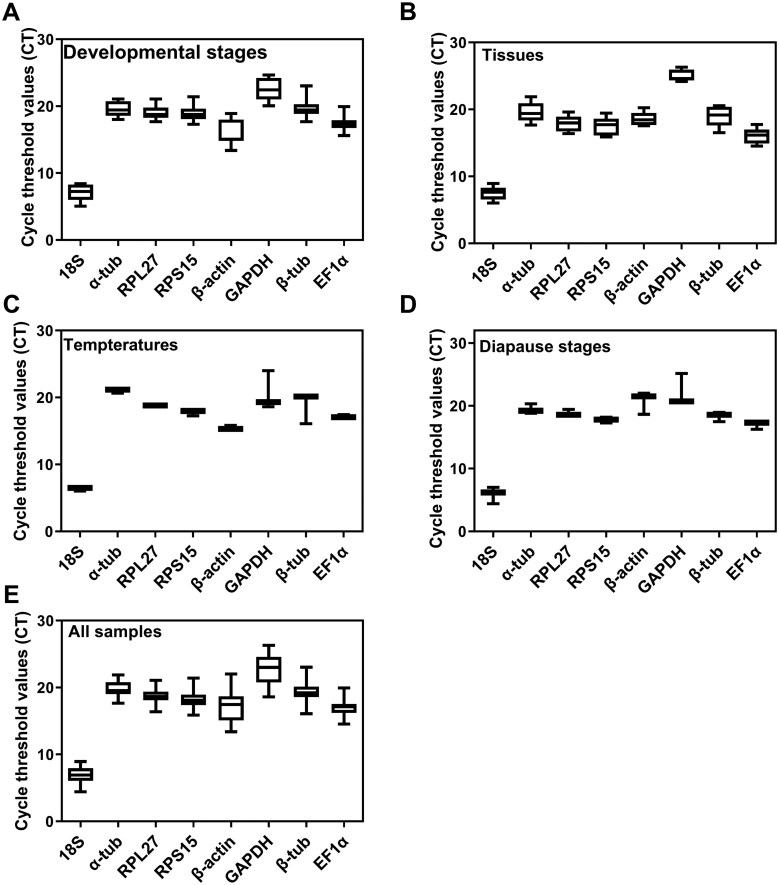
*Ct* value expression profiles of eight candidate reference genes in *Pieris melete* under divergent experimental conditions: A) different developmental stages; B) different tissues; C) different temperatures treatment; D) different diapause stages; and E) all samples. The box plots depict the median (horizontal line), 25% and 75% quartiles (box), and min and max values (whiskers).

The relative abundance of gene expression serves as a primary criterion for selecting reference genes, while variation in *Ct* values is another critical factor for evaluating expression stability. To assess the expression levels and variation of eight candidate reference genes, the mean and SD *Ct* values under different experimental conditions were determined using RT-qPCR and visualized via box plots (Fig. 1). Overall, eight candidate genes had a wide range of the mean *Ct* values ranging from 6.92 to 23.00 cycles and covered all experimental conditions. This indicates that the candidate reference genes exhibited high transcriptional expression levels under varying conditions, meeting the essential criteria for reference gene selection. Notably, transcripts for *18S* exhibited the lowest *Ct* value (6.92), and thus was the most abundantly transcribed across all samples, followed by *EF1α* (17.13), *β-actin* (17.46), *RPS15* (18.08), *RPL27* (18.68), *β-tub* (19.22), *α-tub* (19.54), and *GAPDH* (23.00). Furthermore, the highest variation in expression occurred with *GAPDH* and *β-actin*, while lower variation was observed for the other six genes (*18S*  >  *α-tub*  >  *β-tub* > *RPS15 *>* EF1α* > *RPL27*). The experimental treatments did influence the degree of variability in candidate gene expression. For instance, *GAPDH* showed a high variation range (3.2 to 5.4 cycles) across most experimental treatments but demonstrated minimal variation (1.6 cycles) in tissue samples. Consequently, none of the reference genes exhibited stable expression across all experimental conditions.

### Stability of Candidate Reference Genes Expression across Treatments

To determine the most appropriate reference gene(s) for the four experimental conditions, four algorithms were employed. Then, the overall stability rankings of the reference genes were integrated and analyzed using RefFinder (Table 2).

**Table 2.** Expression stability of the candidate reference genes under different experimental conditions in *Pieris melete*

Treatment	Genes	Δ*Ct*		BestKeeper		NormFinder		geNorm	
		Stability	Rank	Stability	Rank	Stability	Rank	Stability	Rank
Developmental stages	*18S*	2.052	7	1.027	6	1.796	7	1.349	7
	*α-tub*	1.478	5	0.872	3	0.944	5	0.748	5
	*RPL27*	1.186	3	0.841	2	0.342	2	0.306	1
	*RPS15*	1.166	2	0.882	4	0.442	3	0.306	1
	*β-actin*	1.965	6	1.603	8	1.672	6	1.093	6
	*GAPDH*	2.203	8	1.309	7	1.938	8	1.563	8
	*β-tub*	1.322	4	1.011	5	0.840	4	0.506	4
	*EF1α*	1.131	1	0.800	1	0.301	1	0.383	3
Tissues	*18S*	0.970	3	0.728	2	0.457	2	0.460	4
	*α-tub*	1.466	7	1.066	7	1.276	7	0.947	7
	*RPL27*	0.880	1	0.904	5	0.385	1	0.199	1
	*RPS15*	0.984	4	1.051	6	0.627	4	0.199	1
	*β-actin*	0.938	2	0.749	3	0.479	3	0.295	3
	*GAPDH*	1.809	8	0.724	1	1.675	8	1.163	8
	*β-tub*	1.123	5	1.140	8	0.794	6	0.630	5
	*EF1α*	1.132	6	0.866	4	0.693	5	0.787	6
Temperatures	*18S*	0.974	4	0.239	3	0.675	6	0.034	1
	*α-tub*	0.961	3	0.252	4	0.639	4	0.034	1
	*RPL27*	1.016	6	0.032	1	0.643	5	0.234	4
	*RPS15*	0.992	5	0.343	6	0.584	3	0.118	3
	*β-actin*	0.930	2	0.273	5	0.046	1	0.320	6
	*GAPDH*	2.725	8	2.240	8	2.568	8	1.364	8
	*β-tub*	2.390	7	1.804	7	2.143	7	0.911	7
	*EF1α*	0.926	1	0.203	2	0.046	1	0.299	5
Diapause stages	*18S*	1.287	6	0.980	6	0.823	5	0.717	6
	*α-tub*	0.919	1	0.589	5	0.160	1	0.325	3
	*RPL27*	0.926	2	0.383	2	0.230	2	0.268	1
	*RPS15*	0.964	3	0.325	1	0.591	3	0.268	1
	*β-actin*	1.698	7	1.381	7	1.493	7	0.983	7
	*GAPDH*	2.112	8	2.024	8	1.954	8	1.265	8
	*β-tub*	1.039	4	0.562	4	0.739	4	0.369	4
	*EF1α*	1.176	5	0.500	3	1.005	6	0.411	5
All samples	*18S*	1.903	6	0.952	4	1.290	6	1.234	6
	*α-tub*	1.769	5	0.981	5	1.144	5	1.050	5
	*RPL27*	1.485	1	0.743	1	0.661	1	0.495	1
	*RPS15*	1.534	3	0.884	3	0.821	2	0.495	1
	*β-actin*	2.656	7	2.046	8	2.421	8	1.916	8
	*GAPDH*	2.730	8	1.940	7	2.321	7	1.644	7
	*β-tub*	1.720	4	1.121	6	1.067	4	0.865	4
	*EF1α*	1.532	2	0.826	2	0.835	3	0.613	3

The “All samples” category represents a reanalysis of the stability of reference genes by combining the qPCR data from all individual treatment groups and sample types into a single dataset.

### Developmental Stages

**Fig. 2.**
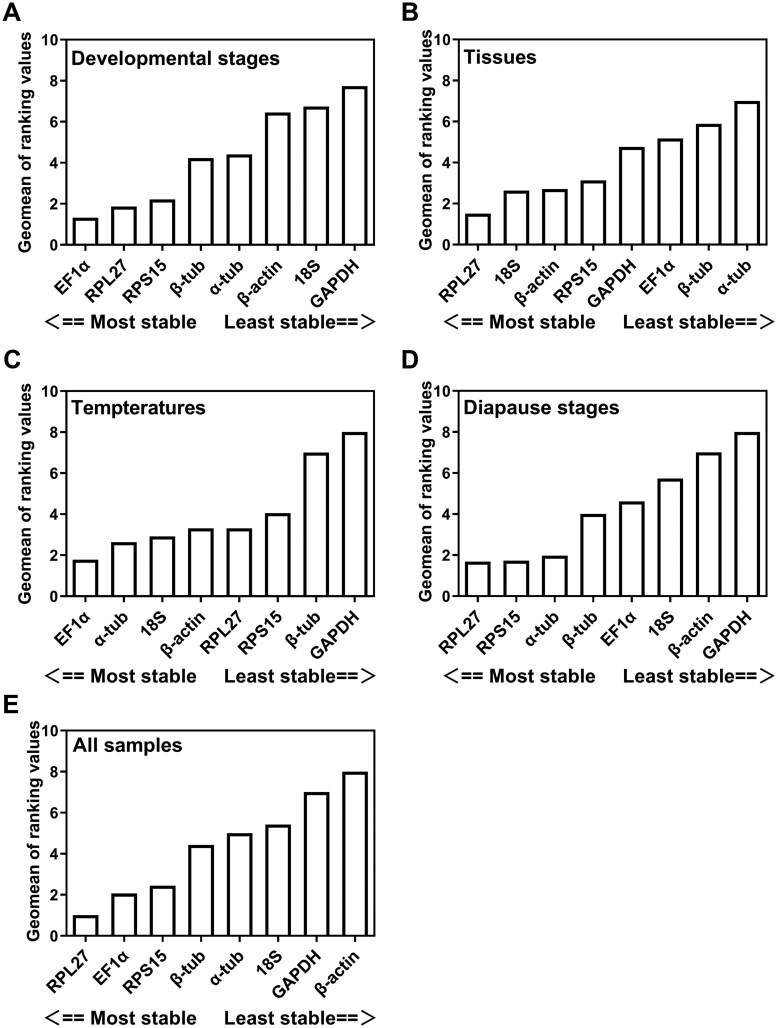
RefFinder-based expression stability of candidate reference genes in *Pieris melete*. The average expression stability of the reference genes was calculated by the Geomean method of RefFinder. A lower Geomean value indicates more stable expression: A) different developmental stages; B) different tissues; C) different temperatures treatment; D) different diapause stages; and E) all samples.

For different developmental stages, the Δ*Ct* method and geNorm analysis identified *EF1α*, *RPL27*, and *RPS15* as the top three stable reference genes. Similarly, the estimation of BestKeeper and NormFinder concluded that *EF1α* and *RPL27* exhibited the highest stability (Table 2). Consistently, all four statistical algorithms indicated that *GAPDH*, *18S*, and *β-actin* were the most unstable reference genes. The comprehensive rankings generated by RefFinder ranked the genes in the following order of stability: *EF1α* > *RPL27 *>* RPS15* > *β-tub*  >  *α-tub*  >  *β-actin* > *18S* > *GAPDH* (Fig. 2).

### Tissues

For the tissue-specific experiment, samples from the entire body showed *RPL27* as the most stable among results from all four programs, except BestKeeper, and correspondingly *GAPDH* as the least stable genes, while it was determined as the most stable reference gene by BestKeeper (Table 2). Besides, *18S* and *β-actin* were determined as the top suitable reference genes after *RPL27*, across template levels using Δ*Ct*, NormFinder, and BestKeeper. According to the results of RefFinder, the stability ranking from the most stable to least stable across different tissues was as follows: *RPL27 *>* 18S  *>  *β-actin* > *RPS15* > *α-tub* > *GAPDH* > *EF1α  *>  *β-tub* (Fig. 2).

### Temperatures

The stability rankings under different temperature stress treatments varied significantly among the four algorithms. *EF1α* and *β-actin* were identified as the most stable genes based on Δ*Ct* method and NormFinder; *EF1α* and *RPL27* were the most stable based on BestKeeper; and the best reference genes from geNorm were *α-tub* and *18S* (Table 2). In contrast, all four programs identified *β-tub* and *GAPDH* as the least and second least stable genes for samples exposed to cold shock, normal temperature and heat shock. Subsequent results of RefFinder ranked EF1α  >  *α-tub* > *18S*  >  *β-actin* > *RPL27 *>* RPS15* > *β-tub* > *GAPDH* in order of most to least stable (Fig. 2).

### Diapause Stages

For diverse diapause stages, the rank order of the reference genes by different software showed similar trends with subtle variation. *α-tub* and *RPL27* were determined to be the top two stable genes by Δ*Ct* method and Normfinder, whereas *RPS15* and *RPL27* were recommended as the top two stable genes using geNorm and BestKeeper. Conversely, *β-actin* and *GAPDH* were deemed the most unstable genes across all algorithms (Table 2). RefFinder analysis demonstrated *RPL27* as the most stable reference gene, followed by *RPS15*, *α-tub*, *β-tub*, *EF1α*, *18S*, *β-actin*, and *GAPDH* (Fig. 2).

### All Samples

To provide a general guideline for the selection of reference genes, the original RT-qPCR data from all tested samples were assembled for evaluation. According to Δ*Ct* value, BestKeeper and NormFinder analyses, the most stably expressed reference genes of *P. melete* were *RPL27*, *EF1α*, and *RPS15* (Table 2). *RPL27* and *RPS15* were stably expressed genes according to the GeNorm analysis. Moreover, all software packages identified *β-actin* as the least stable gene (Fig. 2).

### Optimal Number of Reference Genes for RT-qPCR Normalization

**Fig. 3.**
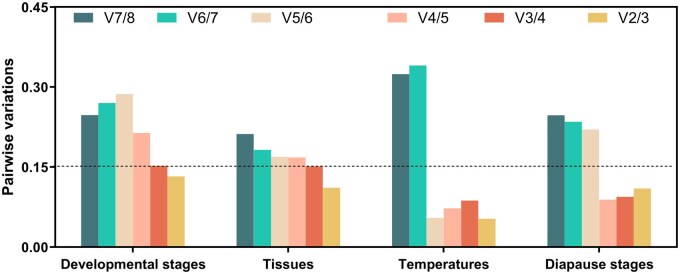
Determination of optimal reference gene number in *Pieris melete* across experimental treatments. The geNorm algorithm calculated sequential pairwise variation ratios (*Vn/n + *1) to establish normalization requirements, where values below the 0.15 threshold indicate stabilization of normalization efficiency at *n* reference genes.

While a single stable reference gene with moderate to high expression levels may suffice for RT-qPCR data normalization, employing multiple validated reference genes is strongly recommended to enhance normalization accuracy (Vandesompele et al. 2002). Based on a comprehensive analysis using RefFinder, for both treatments in this study, *V*_2/3_ value was <0.15 (Fig. 3). Therefore, optimal reference gene combinations were determined for distinct experimental conditions: *RPL27* and *EF1α* for developmental stage comparisons; *RPL27* and *18S* rRNA for larval tissue analyses; *EF1α* and *α-tub* under temperature stress conditions; and *RPL27* with *RPS15* in different diapause stages.

## Discussion

In molecular biology, relative quantification of a target gene—achieved by comparing its expression to that of internal reference genes—allows for sample normalization to account for technical and procedural errors (Pfaffl 2001). However, selecting appropriate reference genes is pivotal to ensuring reliable RT-qPCR data, given that no single gene maintains consistently stable expression across varying developmental stages, tissue types, or environmental stimuli such as temperature fluctuations, nutrient deficiencies, and insecticide exposure (Kang et al. 2017). For this reason, a systematic assessment of reference gene stability is essential prior to initiating experimental work.

*P. melete* is a serious pest for cruciferous mountain plants. With the advancement of high-throughput sequencing technologies, an increasing amount of genetic information has been accumulated for *P. melete* (Wu et al. 2018, Jiang et al. 2021, Miano et al. 2022, Zhang et al. 2022). To explore the biological functions of specific genes in *P. melete*, precise quantification of gene expression is essential, which relies on the identification of stably expressed reference genes. However, to date, no optimal reference gene, or combination of genes, has been identified and validated for this species. In this work, we identified the most stable reference genes for normalizing gene expression across different experimental conditions, including tissue types, developmental stages, temperature treatments, and diapause phases. The findings of this study make a substantial contribution to advancing the genetic research of *P. melete*.

Eight candidate reference genes were selected from the de novo transcriptome assembly, based on commonly utilized reference genes known for their stability in insect studies (Lü et al. 2018). To minimize errors from selecting co-regulated transcripts, candidate genes from different functional categories were chosen and evaluated using four statistical methods: the Δ*Ct* method, BestKeeper, NormFinder, and geNorm. When comparing results across tissues, we observed that the rankings generated by BestKeeper occasionally contradicted those produced by Δ*Ct*, geNorm, and NormFinder (Table 2). These discrepancies are anticipated, as each algorithm is based on distinct statistical frameworks (Guo et al. 2014, Robledo et al. 2014). The discrepancies in ranking orders between the four methods pose challenges in selecting the most suitable reference genes. To overcome this, we employed RefFinder, a tool that combines the results from multiple algorithms to provide a comprehensive ranking of gene stability. RefFinder has been effectively used to assess the stability of reference genes in RT-qPCR studies in lepidoptera species by integrating predictions from various statistical methods (Xie et al. 2012, Wu et al. 2014, Xie et al. 2023). Accurate gene expression quantification using RT-qPCR requires prior validation of reference genes, including an assessment of amplification efficiency (*E*) and the linear dynamic range across different template concentrations. Our primer validation through standard curve analysis demonstrated amplification efficiencies ranging from 93.47% to 106.09%, with correlation coefficients (*R*^2^) exceeding 0.990, thus meeting the technical requirements for RT-qPCR.

Ribosomal proteins (RPs) represent a highly conserved protein family that is essential for ribosome assembly and protein synthesis. Among them, members of the *RPL* and *RPS* gene families are frequently employed as internal reference genes to normalize RT-qPCR data in insects (Lü et al. 2018). For example, RPs exhibit stable expression patterns for various experiments in *S. litura*, *Rhyzopertha dominica*, *Agasicles hygrophila*, and *Ferrisia gilli* (Guo et al. 2021, Bansal et al. 2023, Wu et al. 2024, Xue et al. 2024). Our study identified *RPL27* as the most stable reference gene across developmental stages, tissue types, diapause stages, and all samples. In accordance with our finding, *RPL27* was recommended as the most appropriate reference genes for molecular studies in *Apolygus lucorum* and *M. loreyi* (Luo et al. 2020, Wang et al. 2024). Notably, during diapause, *RPS15* also ranked among the most stable reference genes. This result diverges significantly in *Chrysoperla nipponensis*, whose most reliable gene combinations under adult diapause tissues were *Tub1* and *RpS26e* (Wang et al. 2020). These observations suggest that reference gene stability is influenced by species-specific biological characteristics.

Transcription elongation factors (EFs), another conserved protein family, play a pivotal role in RNA polymerase function and facilitate transcriptional progression through nucleosomes. EFs are generally regarded as stable reference genes under a range of experimental conditions in lepidopteran. Specifically, *EF1α* is widely recognized as one of the 10 most frequently used internal controls for RT-qPCR (Lü et al. 2018). Previous studies have reported its expression stability across developmental stages (Guo et al. 2020), tissue expression (Ma et al. 2021, Kong et al. 2024), sexes (Meng et al. 2022, Wang et al. 2022), different temperatures (Zou et al. 2024), and other treatments (Wang et al. 2022, Yuan et al. 2024). Consistent with these reports, *EF1α* was also found to be one of the most stable genes across developmental stages and different temperatures of *P. melete*. However, *EF1α* was reported to be the least stable gene in total and head tissues between *Sitobion avenae* morphs (Li et al. 2022).

Tubulins, structural components of the cytoskeleton, are essential for maintaining cell shape and integrity and are frequently used as reference genes. *Tub* was reported as the most stable gene in *Paederus fuscipes* under different temperatures (Khan et al. 2022) and in the developmental stages of *Aquatica leii* (Yang et al. 2020). In contrast, it was identified as the least stable gene under temperature and starvation stresses in larvae *S. frugiperda* (Shu et al. 2021). Regardless of these contradictory results, our results revealed that *α-tub* was the most stable gene under temperature stresses in *P. melete*, consistent with previous observations in *Aphis glycines* (Wang et al. 2022).

In *P. melete*, *18S* was identified as the most stable gene across different tissues. It has been validated as a reliable internal control under varying temperature conditions in *Dendroctonus valens* and *R. dominica* (Zheng et al. 2020, Xue et al. 2024), and across developmental stages in *Lasioderma serricorne* and *Tamarixia radiata* (Guo et al. 2020, Zhang et al. 2021). However, it was found to be among the least stable genes under starvation stress in *R. dominica*, reinforcing the necessity of validating reference genes before qPCR-based expression studies (Xue et al. 2024).

While *β-actin* and *GAPDH* have been employed as reference genes in nearly 90% of published insect qPCR studies (Shakeel et al. 2018), their expression stability is often compromised under various experimental treatments (Lü et al. 2018, Luo et al. 2018). In this study, *GAPDH* showed the highest variability across all tested conditions, while *β-actin* displayed stable expression across tissues but varied significantly under other treatments, suggesting that *β-actin* and *GAPDH* are not suitable reference genes for *P. melete* under most experimental conditions. This aligns with previous findings in *Diabrotica virgifera* and *P. xylostella*, where both genes demonstrated unstable expression in specific treatments (Barros Rodrigues 2014, You et al. 2018). Our results for *β-actin* and *GAPDH* further suggest that expression of these commonly used reference genes can be unstable in experimental conditions, and careful evaluation should be undertaken when designing assays.

Historically, although single reference gene has been used for normalization in gene expression studies, while mounting evidence suggests that employing multiple reference genes enhances reliability (Concha et al. 2012). Overuse of reference genes, however, may introduce complexity in data interpretation (Ling and Salvaterra 2011). The pairwise variation (*Vn*/*Vn + *1) method has been widely accepted to determine the minimal number of required reference genes. In our study, all *V*_2/3_ values were below the 0.15 threshold, indicating that two reference genes are sufficient for accurate normalization in *P. melete*, in line with previous recommendations.

In conclusion, our analysis revealed that reference gene rankings varied with experimental conditions, suggesting that expression stability is modulated by both species- and treatment-specific factors. Despite methodological differences among algorithms, consensus was observed for the top-performing reference genes under each condition. Based on these results, we propose a set of optimal reference gene combinations for specific experimental conditions in *P. melete*. These insights are consistent with previous findings in other lepidopteran, emphasizing the importance of validating reference genes based on specific experimental contexts in insect molecular studies.

## Supplementary Material

ieaf108_Supplementary_Data
