# Supplementary material for: Selection and evaluation of reference genes for quantitative real-time polymerase chain reaction normalization in Pieris melete (Lepidoptera, Pieridae)
Source: J Insect Sci. 2025 Dec 22;25(6):ieaf108. doi: 10.1093/jisesa/ieaf108 (PMC12721080; doi:10.1093/jisesa/ieaf108)
Supplement: ieaf108_Supplementary_Data [file ieaf108_supplementary_data.zip › Supplementary material-S1.docx]

**Supplement**

**Tables**

Table S1. Blast results of candidate reference genes for RT-qPCR studies in *Pieris melete*

| Gene | Hit-species | Hit-accession number | Identity (%) | E-value | Locus description |
| --- | --- | --- | --- | --- | --- |
| *α-tub* | *Melipona quadrifasciata* | KOX75272.1 | 99.32 | 0.0 | tubulin alpha-1 chain |
| *β-actin* | *Vanessa tameamea* | XP_026500563.2 | 76.56 | 4e-71 | actin-1-like |
| *EF1a* | *Tribolium castaneum* | XM_047664672.1 | 94.84 | 0.0 | elongation factor 1-alpha 1-like Protein |
| *RPL27* | *Bombyx mori* | NP_001037235.1 | 93.28 | 1e-86 | ribosomal protein L27 |
| *RPS15* | *Bombyx mori* | NP_001037209.1 | 98.64 | 2e-79 | ribosomal protein S15 |
| *18S* | *Pieris rapae* | XR_006751075.1 | 99.94 | 0.0 | small subunit ribosomal RNA |
| *β-tub* | *Maniola hyperantus* | XP_034824109.1 | 92.56 | 0.0 | tubulin beta chain-like |
| *GAPDH* | *Bombyx mori* | NP_001037386.1 | 93.98 | 0.0 | glyceraldehyde-3-phosphate dehydrogenase |

**Figure captions**

Fig. S1. Agarose gel electrophoresis to test primer specificity. Results of RT-qPCR are presented for primer pairs used to amplify candidate reference genes in *Pieris melete.* Details on primers and product sizes are provided in Table 1.

Fig. S2. Melt curve analysis of eight reference genes. A single peak indicates the gene-specific amplification.

**Figures**

**Figure S1.**

**Figure S2.**
