# Supplementary material for: Selection and evaluation of reference genes for quantitative real-time polymerase chain reaction normalization in Pieris melete (Lepidoptera, Pieridae)
Source: J Insect Sci. 2025 Dec 22;25(6):ieaf108. doi: 10.1093/jisesa/ieaf108 (PMC12721080; doi:10.1093/jisesa/ieaf108)
Supplement: ieaf108_Supplementary_Data [file ieaf108_supplementary_data.zip › Supplementary material-S2.docx]

>CL10624.Contig1_All *α-tub*

TTAGTACTCTTCAGCACCTTCGCCTTCTCCCTCAGCAGAGTCCATACCGACCTCTTCGTAGTCCTTCTCAAGAGCTGCCAAGTCCTCACGGGCCTCGGAGAACTCTCCCTCCTCCATACCCTCACCGACGTACCAGTGGACGAAAGCACGCTTGGCGTACATAAGGTCGAACTTGTGGTCCAAACGAGCCCAAGCCTCAGCGATGGCAGTGGTGTTAGACAACATGCACACGGCACGCTGTACCTTGGCCAAGTCACCACCAGGAACAACAGTGGGTGGCTGGTAGTTAATACCGACCTTGAAACCGGTAGGACACCAGTCTACAAACTGAATGGTGCGCTTGGTCTTGATGGTAGCAATGGCAGCGTTTACGTCCTTAGGCACGACGTCACCACGGTACAACATGCAGCAAGCCATGTATTTGCCGTGACGGGGGTCGCATTTTACCATCTGGTTTGCGGGCTCAAAGCAAGCGTTGGTGATTTCGGCAACCGACAACTGTTCATGGTACGCCTTCTCAGCAGAGATGACTGGGGCGTAGGTGACCAACGGGAAGTGGATACGTGGGTAGGGGACCAAGTTGGTCTGGAACTCGGTAAGATCGACGTTGAGTGCGCCGTCGAAACGCAGGGAGGCCGTAATTGAAGATACGATTTGACCGATAAGCCTGTTCAGGTTAGTGTAGGTTGGGCGTTCAATGTCCAAGTTGCGTCTGCAGATGTCGTAGATAGCTTCATTGTCGACCATGAAGGCACAGTCGGAGTGCTCCAGGGTGGTGTGGGTGGTGAGGATGGAGTTGTAGGGTTCAACAACAGCAGTGGAGACTTGGGGAGCTGGGTAGATAGCGAACTCAAGCTTAGACTTCTTTCCGTAGTCCACTGAGAGACGCTCCATAAGAAGAGATGTGAAACCAGATCCAGTGCCTCCTCCGAATGAGTGGAAGATAAGGAAACCCTGAAGACCAGTGCACTGGTCAGCAAGCTTGCGGATTCTGTCAAGGACTAGGTCGACGATTTCCTTTCCGATTGTGTAGTGACCACGCGCGTAGTTGTTGGCAGCATCTTCCTTACCAGTGATAAGTTGTTCTGGATGAAACAACTGTCTGTATGTGCCTGTGCGAACCTCATCAACTACTGTAGGCTCCAAGTCAACAAACACAGCCCTGGGGACGTGCTTGCCGGCTCCCGTCTCACTGAAGAAAGTGTTGAAAGAGTCATCACCACCACCAATGGTTTTGTCTGTGGGCATCTGACCATCAGGCTGGATGCCATGCTCAAGGCAATACAGCTCCCAGCAGGCATTACCGATCTGGACTCCCGCTTGACCAACGTGGACTGAGATACATTCACGCAT

>Unigene2723_All *β-actin*

GAATTTAGTACTTCGAAAAATACTTCGCAGCATTTTATTTTTTCGTCTATGGTTGAAGAATTGCAACAAGCCAACATGACCGCCCTGTCTTCAGGCGCGGCCTTCAGCTCCCTATAGAAAACGTGATGCCATATCCTCTCCATGTTGTCCCAGTGTATTATCTTTCCGTTTACTATGGGACTGCTCAGTTCCAGGTCGTCTATTTTGGCGATCGCATCATCGCCAATAAAGACGTCGTACGGTTCCCGGCCGTAGCTGCCATGTAAATAGCTCGGTCGTCCTACTAACGTTCGGAACATCGATACCGGGTGGTTATCGCATGCAAATCCTGCCTTCATAATGTAACTGCCGTTATCTAATACGATAGCGGGCTTTTCGAACGCCAT

>Unigene18811_All *EF1α*

CTACTTCTTGCCTCCCTTGGTAGCTTTTTCGGCGGCCTTGGTGACCTTTCCACTACCTTCCTTGAAGTTAACGGCCTTGATGACTCCTACAGCCACTGTTTGTCTCATGTCACGGACAGCAAAACGTCCAAGAGGTGGGAATTCCTGGAAGGACTCCACACAGAGGGGCTTGGAAGGAACAAGGATTACAATAGCAGCATCACCAGACTTGATGGATTTAGGGTTGTCTTCTGTTGATTTTCCAGTACGACGGTCTACCTTCTCTTTGATTTCAGCGAATTTGCAAGCAATGTGAGCTGTGTGGCAATCAAGGACTGGGGTGTAACCGTTGGAGATTTGTCCAGGATGGTTCAACACAATGACCTGTGCTGTGAAGTCGGCAGCCCCCTTAGGTGGGTTGCTCTTTGAGTCACCAGCGACGTAACCACGACGCAATTCCTTGACAGAGACGTTCTTTACGTTGAATCCGACATTGTCTCCAGGCACAGCCTCTTGGAGAGCTTCGTGGTGCATTTCAACGGACTTAACTTCAGTGGTGATGTTAGCTGGGGCGAATACGACAATGGTACCAGGCTTGAGGACTCCAGTTTCAACTCTGCCGACGGGCACTGTTCCAATACCACCAATTTTGTATACATCCTGAAGGGGTAAACGTAGGGCCTTGTCAGTGGGACGGGCAGGTGGCAAGATAGCATCAAGAGCCTCAATCAGGCATTTACCTTCAGCCTTGCCTTCCTTACGTTCAACATTCCATCCCTTGAACCAGGGCATCTTGGTTGATGGCTCCAACATGTTGTCTCCGTGCCAGCCAGAAATGGGTACGAAAGCAACGGCAGCTGGGTTGTATCCGATCTTCTTAATGTAAGAGGAAACTTCCTTTTTGATTTCCTCAAAACGAGACTCATTGTAGGGAGGTTCAGTAGAGTCCATCTTGTTGACACCAACAATCAGCTGCTTGACACCAAGCGTGAAAGCGAGAAGGGCGTGTTCACGGGTTTGGCCGTTCTTTGAGATACCAGCTTCAAACTCACCAGTACCGGCGGCAACGATAAGTACGGCACAGTCAGCCTGGGAAGTTCCAGTGATCATGTTCTTGATGAAATCTCTGTGTCCAGGTGCATCAATGATGGTAACGTAGTATTTGCCGGTTTCGAACTTCCAAAGAGCAATGTCAATGGTGATACCACGTTCACGCTCGGCCTTCAGTTTGTCCAAAACCCATGCGTATTTGAAGGAACCCTTACCCATTTCCTGGGCTTCCTTCTCGAACTTCTCGATGGTACGTTTGTCGATACCACCACATTTGTAAATCAAGTGGCCGGTGGTGGTGGACTTGCCGGAGTCGACGTGTCCAATGACGACAATGTTAATGTGAATCTTTTCCTTTCCCAT

>CL7020.Contig1_All *RPL27*

TTAGAACCTAAGCTTCTGGAAGAACCACTTGTTCTTACCACTCTTGTATCTCTCCTCAAAGCGCACTCTTGTGTTGAAGCGTAGCTTCTTGCGTTTTGCTGGGTCTTTTAAGTCCTTAGCACTGAACTTTTCAAAACTGATGTCTAAGGAGTACCTAGTGGGCATCAGGTGGTTGTAGTTAACGACCTTGACGAAAGGCTTGACTTTGGAACGCTTGTGGATTTTGTTTTTGCCCATCCTCTTGTGAACTTTCCTGGGGTATCTATCAATACCGGCGAGAAAGGCGTGAGCGTATGGCTTTTCAGCTGTTCCCTCGTCATAGGTCTTAACGACGATAGCTTTACGCCCCGCGTACCGGCCACTCAGGACCAGCACCACTTTACCAGGTTTCATAATCTTACCCAT

>Unigene4152_All *RPS15*

TTTTTTTTTTTTTTTTTTTTTATTGTTTACAATCATTTATTGTTGCAAACACTTTTTTAATAGCTTAAACCTAGGAGAAAGACATCCTACTTAAGAGGAATGAATCTGGAGCTGTGGGTGGCACCAATACCAGGTCTACCGTGCTTGACAGGCTTGTATGTGACTGAAAATTCTCCAAGATAGTGGCCAATCATCTCAGGCTTGATTTCAACCTGGTTGAAAGTCTTTCCATTGTAAATCCCCACAATTGATCCGACCATTTCTGGGACTATGATCATGTTCCGTAAATGTGTCTTTACAATTTCGGGTTTTTCATTTGGTGGTGCTTCTTTCTTCGCACGGCGCAGTTTCTTTACCAGCGCCATTGGTTTCCTCTTAAGACCACGCGCGAATCGCCGGCGAGCGCGGGCATGCATTAACTCCATGAGTTGTTCATTGGGCATATCCAGAAGCTGATCTAAGTCAACCCCTCGGAAGGTAAACTTCCTGAAGGTACGCTTCTTCTTCAAAGTTTCGTCGACCTCAGCCATGTTTCAACACAAATGACAAAACGAAAAGAAATGTATGACGTTGACATCCTACAATTTTGGATTTGGCATATCATCTGTCATTTATCATTCCACATTTGACAACGCTGTTTGGCACTCAG

>CL554. 1881.Contig1_All *18S rRNA*

AGCCATGCATGTCTCAGTGCAAGCCGTATTAAGGCGATACCGCGAATGGCTCAATATATCAGTTTTGGTTCCTTAGATCTTACTCAGTTACTTGGATAACTGTGGTAATTCTAGAGCTAATACATGCAATCAGAACTCTGACCAGTGATGGGATGAGTGCTTTTATTAGATCAAAACCAATCGGCGGAGGGCCTAGCGTCCGAAGTCGTTAATTTTGATGAATCTGGATAACTTTTGCCGATCGCACGGTCCAGTACCGGCGACGCATCTTTCAAATGTCTGCCTTATCAACTTTCGATGGTAGTTTCTGCGACTACCATGGTTGTCACGGGTAACGGGGAATCAGGGTTCGATTCCGGAGAGGGAGCCTGAGAAACGGCTACCACATCCAAGGAAGGCAGCAGGCGCGCAAATTACCCACTCCCGGCACGGGGAGGTAGTGACGAAAAATAACGATACGGGACTCTTACGAGGCCTCGTAATCGGAATGAGTACACTTTAAATATTTTAACGAGGAACAATTGGAGGGCAAGTCTGGTGCCAGCAGCCGCGGTAATTCCAGCTCCAATAGCGTATACTAAAATTGTTGCGGTTAAAAAGCTCGTAGTTGCATTTGTGCGCCGCGCTGTCGGTGCACCGCATCCGCGGTGATACTGACACGTCTGCGGAGCATATCGTCGGTGAGCCGGCGGTAAAACGCCGGTTCAATATCAAAATCCTATCGCGGTGCTCTTCGGTGAGTGTCGAGGTGGGCCGACAATTTTACTTTGAACAAATTAGAGTGCTCAAAGCGGGCTCAAAATGCCGCTTGAATATTTCGTGCATGGAATAATAGAATATGATCTCGGTTCTATTTTGTTGGTTTTCAGAACTCCGAGGTAATGATTAATAGGGATAACTGGGGGCATTCGTATTGCGACGTTAGAGGTGAAATTCTTGGATCGTCGCAAGACGAACATCAGCGAAAGCATTTGCCAAAGGTGTTTTCATCAATCAAGAACGAAAGTTAGAGGTTCGAAGGCGATTAGATACCGCCCTAGTTCTAACCGTAAATATGTCATCTAGCGATCCGCCGACGTTACTACAATGGCTCGGCGGGCAGCTTCCGGGAAACCAAAGATTTTGGACTCCGGGGGGAGTATGGTTGCAAAGCTGAAACTTAAAGGAATTGACGGAAGGGCACCACCAGGAGTGGAGCCTGCGGCTTAATTTGACTCAACACGGGAAATCTCACCAGGCCCGGACACCGGAAGGATTGACAGATTAACAGCTCTTTCTTGATTCGGTGGGTGGTGGTGCATGGCCGTTCTTAGTTGGTGGAGCGATTTGTCTGGTTAATTCCGGTAACGAACGAGACTCTAGCCTGCTAAATAGGCGTCGTCATTCAAGGTGTGCGCGACTCTCGGGGAGCGCAACTCACTGGCGACGTATTAAAATTCTTCTTAGAGGGACCGGCGGCTTCGAGCCGCACGAGATTGAGCAATAACAGGTCTGTGATGCCCTTAGATGTCCTGGGCCGCACGCGCGCTACACTGAAGGAATCAACATGTTCTCCCTGGCCTAGAGGCCCGGGCAACCCGTTGAAACTCCTTCGTGCTGGGGATTGGGGTTTGCAATTATCCCCCATAAACGAGGAATTCCTAGTAAGCGCGAGTCATAAGCTCGCGTTGATTACGTCCCTGCCCTTTGTACACACCGCCCGTCGCTACTACCGATTGAATGATTTAGTGAGGTCTTCGGACCGACACGCGGTGGCCTCACGGCCGTCGGCGTTGCTGGGAAGT

>Unigene7971_All *β-tub*

TTAAACGTCACTTCCATCATTAGGGACTGTTGTTTCCTCTTCCTCAAACTCTTGGTCTTCAATGGTGGCGTCTTGATACTGCTGGTATTCGGATATCAAATCACTAAGGTTATTATCCGCTTCCGTAAAATCAGCTTCATCCATACCTTCACCTGTATACCAGTGTACAAAAGCTTTCCTTCGAAACATAGAAGAAAACTGCTCAGCAATTCGTTTAAATAATTCCTGAATAGCTGTGGTATTGCCTATGAAAGTTGCAGACATTTTAAGACCTCTAGGAGGTATATCGCAAACAGCGGTCTTAACATTACTGGGTATCCATTCAACAAAGTAATCCTTATTCTTGTTTTGAATATTCAGCATTTGTTCATCAACTTCTTTCATGGACATTCTGCCTCTGAAGACCGCAGCTACGGTTAGATATCTCCCGTGGCGTGGATCACATGCTGCCATCATATTTTTGGCATCAAACATCTGCTTTGTAAGTTCTGGGACGGTGAGTGCTCGATATTTTTGGACTCCTCTAGACGTAAGCGGCGCAAAACCGGGCATGAAGAAATGTAACCGCGGAAATGGAACCATATTAACTGCCAGTTTTCGCAAATCAGCGTTCAGCTGTCCGGGAAATCTTAAACAAGTTGTAACACCGGACATCGTTGCTGAAACTAAATGATTTAAGTCGCCATAAGTTGGGGTCTGTAACCGAAGTGTTCTAAAACAAATATCATATAAAGCTTCATTATCAATACAGAAGGATTCATCAGAATTTTCGATAAGTTGGTTCAATGCCAATGTAGCATTGTATGGCTCTACAACAGTATCCGAAACTTTAGGACTAGGAACAACAGAGAAAGTTAGAATAATCCTGTCAGGGTATTCCTCCCTCAATTGGGACAGTATTAAGGTTCCTAATCCAGATCCAGTGCCTCCTCCAATTGAATGGACAACTTGGAATCCTTGTAAACAGTCGCAGCCTTCCGCTTCTTTTCTAACAACGTCAAGGACGGAATCGAGCAAATCTGCTCCTTCTGTGAAGTATCCCTTAGCATAGTTATTGCCTGCACCAGTGCTCCCGTAGACAATATTATCAGGACGAAAGATTTGACCGTATGGGCCGGATCGTAAGGAGTCCATGGTACCAGGTTCTAAATCAACTAAAACGGCTCTCGGCACATATTTGCCACCAGCACCTTCATTGTAATACACGTTGATACGTTCCAACTGAAGATCAGAATCCCCAGAGTAGCAACCATTTGGATCTATTCCATGCTCATCTGATATCACCTCCCAAAACTTCGAACCAATTTGGTTACCACATCTACCAACTTGTACATGAACAATTTCTCTCAT

>Unigene5889_All *GAPDH*

ATGTCGAAAATCGGTATCAACGGATTTGGACGTATTGGACGTCTGGTGCTCCGTGCTTCAGTGGAAAAGGGAGCCAAGGTTGTTGCTATCAATGACCCCTTCATTGGTTTGGATTACATGGTCTACCTTTTCAAGTACGACTCCACTCATGGACGTTTTAAGGGCACAGTTGAGGCTGCTGATGGATGCCTAGTAGTTAACGGAAACAAGATTGCTGTCTTCTCTGAGAGAGACCCTAAAGCTATTCCCTGGGGAAAGGCTGGAGCTGAATATGTTGTTGAGTCCACTGGTGTGTTTACCACCATTGATAAGGCATCTGCTCACATTGAAGGTGGAGCCAAGAAGGTCATCATCTCTGCTCCTAGTGCTGATGCCCCCATGTTTGTAGTAGGTGTAAACCATGAAACCTACGATCCATCCTTCAAAGTCATCTCTAATGCTTCCTGCACAACTAACTGTCTTGCTCCTCTTGCTAAAGTTATCCATGATAACTTTGAAATTGTTGAGGGTCTTATGACCACAGTTCATGCTACTACAGCTACCCAAAAGACAGTTGATGGCCCTTCAGGAAAATTGTGGCGTGATGGTCGTGGTGCTCAACAAAACATTATTCCAGCGGCTACTGGCGCAGCTAAGGCTGTCGGCAAAGTAATCCCTGCTCTTAATGGCAAACTCACTGGAATGGCATTCCGGGTTCCAGTTCCCAATGTCTCTGTTGTTGATTTGACAGTCCGCCTAGGCAAGCCTGCTAGCTATGATGCCATTAAGCAAAAGGTTAAGGAGGCATCTGAAGGACCCCTCAAGGGAATTCTTGGTTACACTGAGGACCAAGTTGTATCAACTGACTTTGTTGGTGATACTCACTCTTCAATCTTTGATGCTGCTGCCGGTATCTCTCTAAATGACAACTTTGTTAAACTTATCAGCTGGTATGACAATGAATATGGTTACTCCAGCCGTGTCATTGACCTTATCAAGTACATCCAAACCAGAGATTAA
