# Supplementary figures and images for: Selection and evaluation of reference genes for quantitative real-time polymerase chain reaction normalization in Pieris melete (Lepidoptera, Pieridae)
Source: J Insect Sci. 2025 Dec 22;25(6):ieaf108. doi: 10.1093/jisesa/ieaf108 (PMC12721080; doi:10.1093/jisesa/ieaf108)

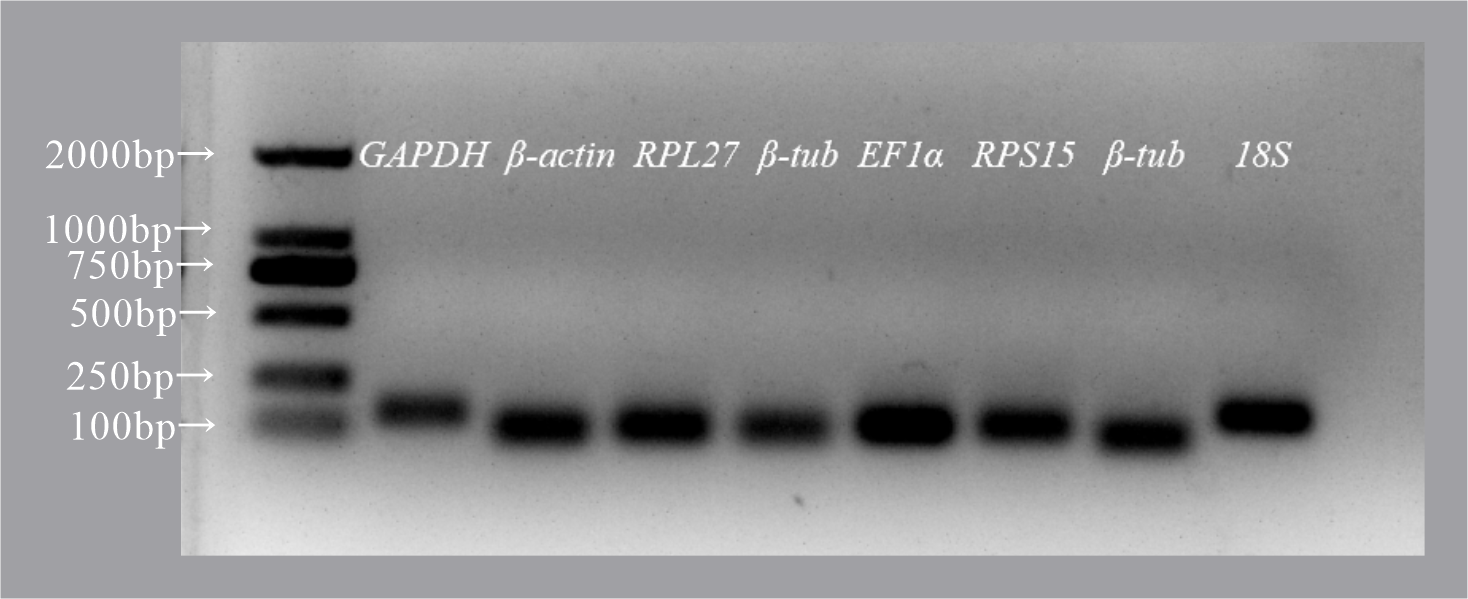

Supplement: ieaf108_Supplementary_Data [file ieaf108_supplementary_data.zip › Figure S1.tif]

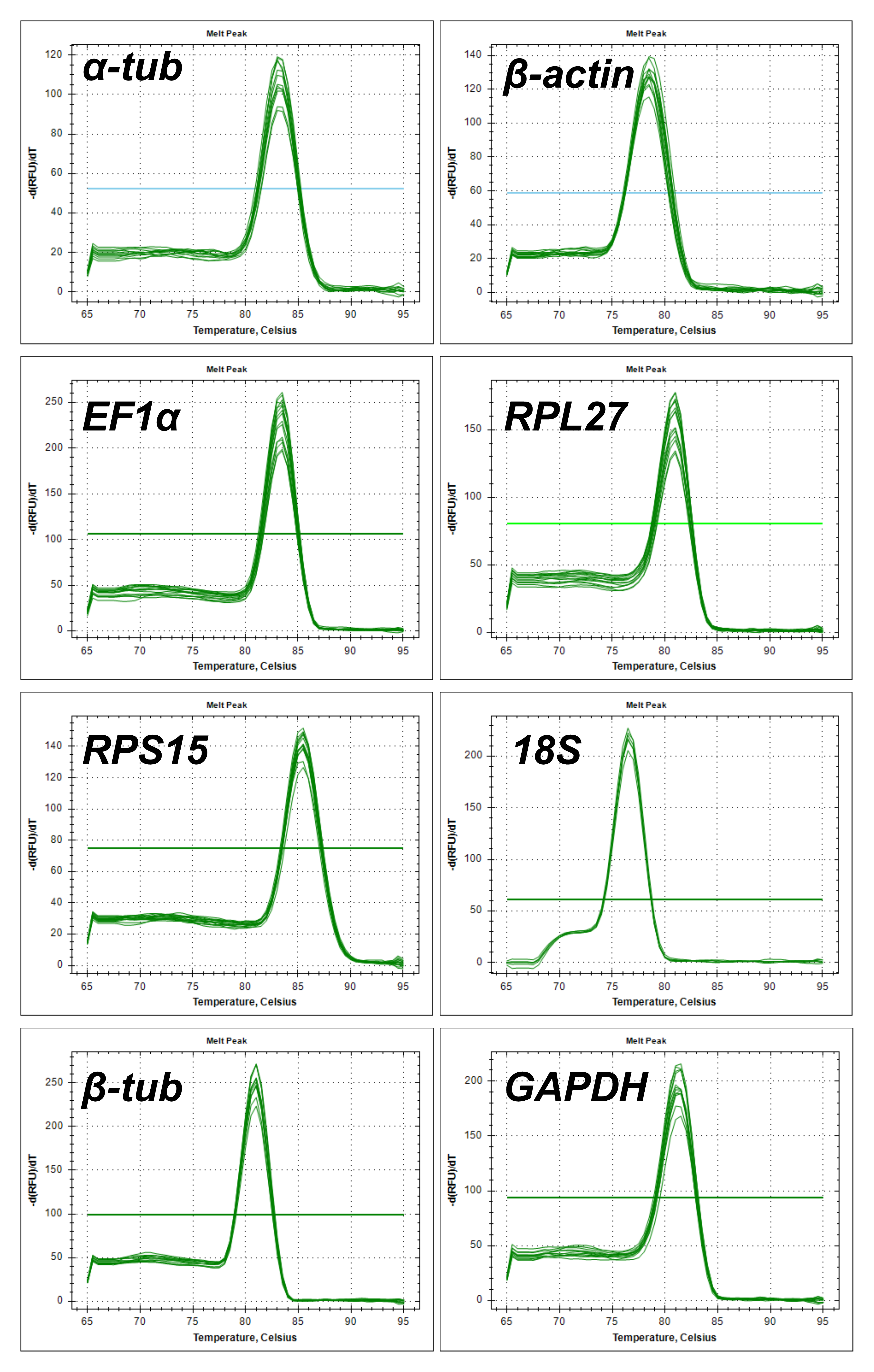

Supplement: ieaf108_Supplementary_Data [file ieaf108_supplementary_data.zip › Figure S2.tif]
